# Illuminating otoliths: New insights for life history of *Balistes* triggerfishes

**DOI:** 10.1111/jfb.15233

**Published:** 2022-10-18

**Authors:** Virginia R. Shervette, Jesús M. Rivera Hernández

**Affiliations:** ^1^ Fish/Fisheries Conservation Lab, Department of Biology/Geology University of South Carolina Aiken Aiken South Carolina USA; ^2^ Marine Sciences, SEOE University of South Carolina Columbia South Carolina USA

**Keywords:** grey triggerfish, life‐history strategy, queen triggerfish, reef fish

## Abstract

Our understanding of fish life‐history strategies is informed by key biological processes, such as growth, survival/mortality, recruitment and sexual maturation, used to characterize fish stocks (populations). Characterizing the life‐history traits of fish populations requires the application of accurate age estimation for managed species. Grey triggerfish *Balistes capriscus* and queen triggerfish *Balistes vetula* are important reef‐associated species for commercial and recreational fisheries in the Atlantic Ocean. Both species exhibit a unique reproductive strategy for large‐bodied fisheries‐targeted reef fishes in that they are nesting benthic spawners and invest substantial energy in defence and care of their benthic nests and fertilized eggs. Until recently, our understanding of the life‐history strategies of triggerfishes assumed the main method used to obtain age estimates, increments counted from thin sections of the first dorsal spine, provided an accurate characterization of population age‐based parameters. However, results from bomb radiocarbon validation studies on the two *Balistes* species demonstrated that spines do not provide accurate ages, but sagittal otoliths do. The main goal of the current study was to provide an updated understanding for triggerfish life‐history strategies by using otolith‐based age estimates to characterize population age structure and growth for grey triggerfish and queen triggerfish from waters of the south‐eastern U.S. Atlantic. The current study is the first to report on sex‐specific age and growth information for grey triggerfish using the Δ^14^C‐validated otolith‐based age estimation method and the results indicate that the previous characterization of *Balistes* species as exhibiting moderately rapid growth and as relatively short‐lived, based on spine‐derived age estimates, are flawed. Otolith‐based ages indicated that grey triggerfish and queen triggerfish are moderately slow‐growing and long‐lived species, attaining maximum ages of 21 and 40 years, respectively. Management efforts for triggerfishes should evaluate these new insights and incorporate the results of otolith‐based age estimation into future population monitoring efforts.

## 1 INTRODUCTION

Our understanding of life‐history strategies of fishes is informed by key biological processes such as growth, survival/mortality, recruitment and sexual maturation that are used to characterize fish stocks (populations). Accurate age estimation for fisheries species is a critical component of ensuring sustainable fisheries management (Beamish & McFarlane, 1983; Campana, 2001). This is because in the assessment of fisheries species, population age‐based parameters, such as longevity, age at sexual maturity, age at sexual transition for sequential hermaphroditic species, growth rate, mortality, age‐specific reproductive output and lifetime reproductive output, are important in understanding overall life‐history strategies of managed species (Beverton, 1998; King & McFarlane, 2003; Lorenzen & Enberg, 2002; Winemiller, 2005).

Triggerfishes (Balistidae) contribute to fisheries across the Atlantic and Indo‐Pacific Oceans (Aggrey‐Fynn, 2013; Albuquerque *et al*., 2011; Allman *et al*., 2018; Barroso‐Soto *et al*., 2007; Matos‐Caraballo, 2018). In the Atlantic, grey triggerfish *Balistes capriscus* Gmelin, 1789, is an important component of commercial and recreational fisheries in the waters of South America (Bernardes, 2002), Central America (Castro‐Pérez *et al*., 2018), western Africa (Ofori‐Danson, 1989; Shervette *et al*., 2021), the Mediterranean (İşmen *et al*., 2004; Kacem & Neifar, 2014) and North America (Allman *et al*., 2018; Burton *et al*., 2015). Another *Balistes* species, queen triggerfish *B. vetula* Linnaeus, 1758, also contributes to fisheries landings in the Atlantic waters of Brazil (Albuquerque *et al*., 2011; Freitas Netto & Madeira di Beneditto, 2010), the Caribbean (Aiken, 1983; Matos‐Caraballo, 2018; Rivera Hernández *et al*., 2019) and the south‐eastern United States (SEUS) waters (Shervette & Rivera Hernández, 2022a; Stevens *et al*., 2019).

*B. capriscus* and *B. vetula* co‐occur throughout much of their ranges in the western Atlantic but appear to vary from each other in their geographic patterns of abundance. In the northern hemisphere of the Atlantic both species are strongly associated with shelf and slope habitats characterized by hard‐bottom and reef‐like structures (Garcia‐Sais, 2010; García‐Sais *et al*., 2007; Glasgow *et al*., 2021; Sedberry & Van Dolah, 1984; Shervette & Rivera Hernández, 2022b). *B. capriscus* occur with greatest abundance at higher latitudes of the shared range, from as far north as waters offshore of North Carolina, United States, down through much of the waters off Florida (Glasgow *et al*., 2021; Kellison & Sedberry, 1998; Muhling *et al*., 2014; Sedberry *et al*., 1998, 2006). *B. capriscus* also occurs at high abundance in the northern Gulf of Mexico (GOM) (Allman *et al*., 2018). *B. vetula* occurs at higher abundance in the lower latitudes of its range, including waters throughout the Caribbean Sea (Robertson & Van Tassell, 2019).

*B. capriscus* and *B. vetula* are gonochoristic species that exhibit a unique reproductive strategy compared to other large‐bodied fisheries‐targeted reef fishes. Both species are nesting benthic spawners that utilize nesting grounds associated with coral reef habitats (Shervette & Rivera Hernández, 2022b; Simmons & Szedlmayer, 2012). *B. capriscus* spawning occurs from late April to early September in waters off North Carolina through north Florida (Kelly‐Stormer *et al*., 2017) and from late May to August in waters of the northern GOM (Ingram, 2001; Lee, 2019). *B. vetula* spawning occurs in the north Caribbean over a longer time period compared to grey triggerfish, starting as early as December and extends through August (Rivera Hernández *et al*., 2019).

Until recently, our understanding of the general life‐history strategy of *Balistes* triggerfish species was based on the assumption that the method used to obtain age estimates, increments counted from thin sections of the first dorsal spine, provided an accurate characterization of population age‐based parameters (Albuquerque *et al*., 2011; Allman *et al*., 2018; Burton *et al*., 2015; Manooch & Drennon, 1987). From spine‐based age estimates, *Balistes* species were thought to exhibit moderately rapid growth (Aiken, 1983; Allman *et al*., 2018; Kelly‐Stormer *et al*., 2017), reach sexual maturity within the first 2 years of life (Aiken, 1983; Ingram, 2001; Moore, 2001) and were relatively short‐lived, attaining maximum ages of 14–15 years (Albuquerque *et al*., 2011; Allman *et al*., 2018; Burton *et al*., 2015; Johnson & Saloman, 1984). However, recent age estimation validation studies utilizing regional patterns of bomb radiocarbon concluded that the first dorsal spine does not provide accurate age estimates for *Balistes* species (Patterson *et al*., 2019; Shervette & Rivera Hernández, 2022a). A study on *B. vetula* from the north Caribbean validated the accuracy of otolith‐based age estimation and demonstrated that spine‐based age estimates resulted in an erroneous characterization of population age structure, growth and longevity compared to the results from otolith‐based ages (Shervette & Rivera Hernández, 2022a). Our new understanding of the life‐history strategy of this species is that *B. vetula* is characterized by moderately slow growth and is long‐lived.

For *B. capriscus*, results from radiocarbon validation efforts indicated that, similar to *B. vetula*, the first dorsal spine does not provide accurate age estimates, but otoliths do (Patterson *et al*., 2019). The next important step in updating the understanding of the general life‐history strategy of triggerfish species is to use otolith‐based age estimates to document population age structure and growth for regional contingents of *B. capriscus* and expand on *B. vetula* by characterizing the age and growth of this species outside of the north Caribbean region. Therefore, the overall goals of this study were two‐fold. The first goal was to provide an updated understanding of the life‐history strategy of *B. capriscus* by using otolith age estimates to describe age‐based population parameters. The second goal was to document the age and growth of *B. vetula* from waters of the SEUS. The specific objectives were to (1) determine the timing of opaque zone formation in triggerfish otoliths, (2) describe population age structure and growth for the two *Balistes* species using samples from the SEUS and (3) compare size‐at‐age between grey triggerfish males and females.

## 2 MATERIALS AND METHODS

### 2.1 Ethics statement

Fish samples obtained by the authors of this study and reported on here were collected and handled in strict accordance within the guidelines of the U.S. Government Principles for the Utilization and Care of Vertebrate Animals Used in Testing, Research and Training (https://olaw.nih.gov/sites/default/files/PHSPolicyLabAnimals.pdf). This research was conducted under USCA IACUC protocol #053012‐BIO‐04.

### 2.2 Study area

Samples for this study were collected from waters off the coasts of North Carolina and South Carolina, United States. This region of the Atlantic is characterized by a wide shelf, extending up to 145 km from the shore. In this region, *B. capriscus* has a relatively broad across‐shelf distribution (Glasgow *et al*., 2021), occurring with moderate to high frequency in the mid‐shelf (20–30 m depths), outer‐shelf (30–50 m depths) and shelf‐edge (50–100 m depths) zones (Muhling *et al*., 2014; Sedberry *et al*., 1998, 2006; Sedberry & Van Dolah, 1984). In the mid‐ and outer‐shelf zones, emergent hard bottom and rock outcrops provide low‐profile three‐dimensional structural complexity that is enhanced by gorgonian corals and sponges (Muhling *et al*., 2014). Artificial reefs and wrecks occur intermittently throughout the study area and provide additional structurally complex habitat for triggerfishes and other fisheries species targeted by commercial and recreational fishing (Kellison & Sedberry, 1998).

*B. vetula* is less common in this region and a dearth of published information exists on this species' habitat associations in SEUS waters. Video surveys conducted in the SEUS from 2015 to 2017 documented a 5.4% frequency of occurrence for queen triggerfish compared to 45.6% for *B. capriscus* (Bacheler *et al*., 2019). In the waters of south‐west Florida, a survey of coral and fish assemblages associated with Pulley Ridge (located in waters of the south‐west corner of Florida) reported *B. vetula* occurred in rock rubble habitat at depths exceeding 60 m (Harter *et al*., 2008). Several studies from the Caribbean reported that adult *B. vetula* are associated with coral reef ecosystem habitats that occur in deeper shelf and shelf edge zones (Garcia‐Sais, 2010; García‐Sais *et al*., 2007; Shervette & Rivera Hernández, 2022b). Diver surveys in Saba Bank documented adults associated with the whole spectrum of coral reef ecosystem strata, but were most abundant in the outer reef flat zone characterized by hard bottom/pavement and a submerged inner reef flat zone with low relief pavement and scattered rubble (Debrot *et al*., 2020; Toller, 2007). *B. vetula* in the current study were caught as incidental catch in deeper shelf waters (>45 m) and at shelf edge sites where commercial fishers were targeting large groupers, snappers and tilefishes.

### 2.3 Sample collection, processing and analyses

*B. capriscus* were collected in 2012–2021 from fisheries‐independent (FI) and fisheries‐dependent (FD) sources. *B. vetula* were collected in 2013–2021 from fisheries‐dependent sources (Table 1). Fish from FI sources included samples obtained using chevron traps as described previously in Kelly‐Stormer *et al*. (2017) and small juvenile fish collected with dipnets from floating sargassum rafts. *B. capriscus* and *B. vetula* FD samples were obtained directly from commercial and recreational fishers at the dock shortly after their return to shore and filleting. A few FD *B. vetula* samples were purchased whole from commercial fishers (Table 1).

**TABLE 1:** Summary results for *Balistes* triggerfish sample collection from waters of the south‐eastern United States

	Number of samples	Size range (mean; s.d.) *L*_f_ (mm)	Age range (mean; s.d.) (years)
*Balistes capriscus*			
All samples	1044	25–571 (363; 61)	0–21 (5.9; 2.3)
Male	502	26–571 (376; 63)	0–17 (5.8; 2.0)
Female	510	25–483 (349; 55)	0–20 (6.1; 2.3)
Fisheries‐independent	44	25–461 (214; 148)	0–10 (2.7; 2.6)
Male	20	26–461 (204; 153)	0–6 (2.2; 2.2)
Female	24	25–381 (223; 147)	0–10 (3.2; 2.8)
Fisheries‐dependent	1000	231–571 (369; 43)	3–21 (6.1; 2.2)
Male	482	293–571 (383; 44)	3–17 (5.9; 1.9)
Female	486	231–483 (355; 36)	3–20 (6.3; 2.2)
*Balistes vetula*			
Fisheries‐dependent	27	355–560 (453; 50)	8–40 (16.0; 7.3)
Male	16	380–560 (469; 53)	8–30 (16.2; 6.7)
Female	11	355–480 (431; 37)	8–40 (15.7; 8.5)

*Note*: Sex of samples was determined *via* histology. ‘All samples’ and ‘Fisheries‐dependent’ for *Balistes capriscus* included 32 individuals of unknown sex that were excluded in summary information for males and females. *L*_f_, fork length; s.d., standard deviation.

**FIGURE 1:**
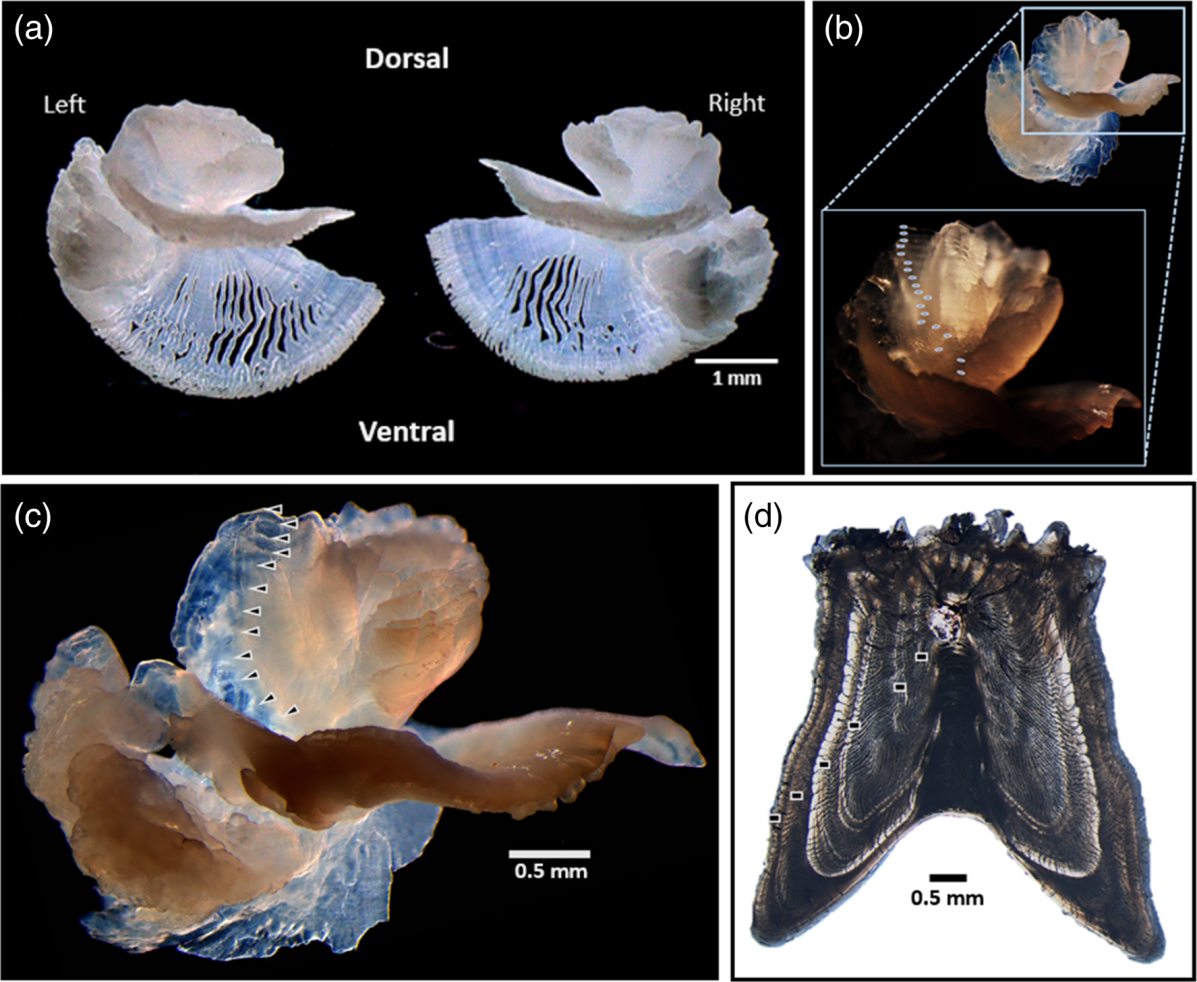
Digital images of *Balistes* otoliths and a dorsal spine section. (a) Intact left and right *Balistes vetula* sagittal otoliths. (b) Left sagitta from a 20‐year‐old *B. capriscus* caught offshore SC viewed using reflected light (upper right) and a zoomed in portion of the same otolith illuminated using concentrated reflected light with a fibre optic cable. To visualize the first opaque zone, the dorsal rim of the otolith must be gently angled towards the reader to see into the cauda area obscured by the ventral margin of the sulcular grove. (c, d) The left sagittal otolith with triangles indicating 12 opaque zones (c) and the first dorsal spine section with rectangles indicating six translucent zones (d), from the same 450 mm female *B. capriscus*

All fish samples were kept on ice until processing occurred. Fish were measured for size (standard length: *L*_s_, fork length: *L*_f_, total length: *L*_t_) to the nearest millimetre. Fish obtained whole were weighed (g). Whenever possible for FD samples and for all FI samples (including small, juvenile fish collected from sargassum), gonads were collected and preserved for histological processing to determine sex following the methods described in Rivera Hernández *et al*. (2019). Sagittal otoliths (Figure 1a) were carefully extracted following the methods described in Rivera Hernández and Shervette (2022), and saved for age estimation.

The Δ^14^C‐validated otolith age estimation protocol for *B. capriscus* and *B. vetula* (Rivera Hernández & Shervette, 2022) was used to obtain ages for samples from both species *via* enumeration of otolith opaque zones (Figure 1b,c). Briefly, sagitta were read whole, submerged in water against a black background with a stereo microscope at a magnification range of 20–40× (Supporting Information Figure S1). This age estimation protocol for triggerfish sagitta includes the use of concentrated light to more effectively illuminate the otolith *via* a fibre optic cable (this allows the reader to control light intensity and angle such that opaque zones appear to glow); each opaque zone present was counted (Shervette *et al*., 2021; Shervette & Rivera Hernández, 2022a). Sagittal otoliths from each fish were read blind (with no knowledge of fish size, date‐of‐collection or sex) by an experienced primary reader. A second experienced reader read (independently and blind) a subset of the otoliths. Percentage agreement and average percentage error (APE) were calculated to assess between reader precision. Samples for which reader disagreement of opaque zone counts occurred were re‐examined simultaneously by both readers and a consensus age estimate was obtained (Shervette *et al*., 2021; Shervette & Rivera Hernández, 2022a). The primary reader noted for each otolith the location of the last opaque zone relative to the otolith edge. For *B. capriscus*, the monthly proportion of otoliths with opaque zones on the edge was plotted to determine the annual periodicity of otolith opaque zone formation (Labropoulou & Papaconstantinou, 2000; Shervette *et al*., 2021; Shervette & Rivera Hernández, 2022a). This information was combined with the peak spawning period (May–July; Kelly‐Stormer *et al*., 2017), which enabled the establishment of an estimated birthdate (1 June) so that fractional age could be computed for each *B. capriscus* sample with an otolith age estimate (see the Results section for detailed criteria developed from edge analysis results).

For *B. capriscus* samples, a two‐factor ANOVA was used to test the effect of sex on estimated size‐at‐age for ages 4–10, the most prevalent age classes in the dataset. The dependent variable for this was *L*_f_; the independent variables were age class and sex (male versus female). Statistical analyses were conducted in SPSS (IBM Corp. 2012) and the results were considered significant at *P* values less than 0.05. If the assumptions for statistical tests were not met, then data were log transformed.

For size‐at‐age data, separate von Bertalanffy growth functions (VBGF) were fit to estimated ages for the following groups: *B. capriscus* all samples combined, *B. capriscus* males, *B. capriscus* females and *B. vetula* all samples combined. VBGF were computed using the least squares method with the solver function in Microsoft Excel (Haddon, 2011). A lack of juvenile *B. vetula* in the SEUS collections necessitated the use of a fixed *t*_0_ value (−0.585) that was previously computed for this species in a recent otolith‐based age and growth study (Shervette & Rivera Hernández, 2022b).

## 3 RESULTS

**FIGURE 2:**
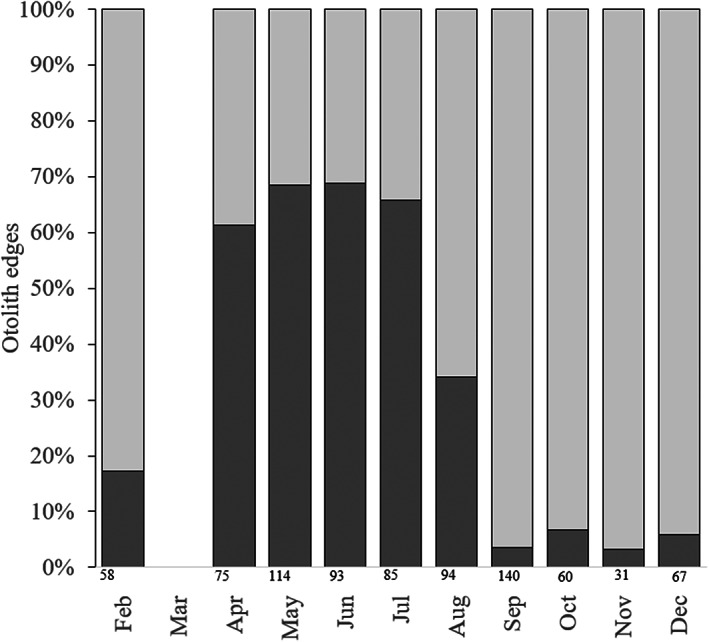
Bar graph indicating the monthly percentage of south‐eastern United States *Balistes capriscus* sagittal otoliths with opaque and translucent zones on the otolith edge. Numbers below each bar indicate the total number of samples within a month with edge information. (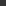) Opaque and (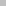) translucent

A total of 1044 grey triggerfish and 27 queen triggerfish samples were processed for otolith‐based age estimates (Table 1). *B. capriscus* ranged in size from 25–571 mm *L*_f_ and in age from 0–21 years; *B. vetula* ranged in size from 355–560 mm *L*_f_ and in age from 8–40 years (Table 1). *B. capriscus* were obtained from all months except January and March with monthly sample numbers ranging from a low of 31 for November to a high of 140 for September. *B. vetula* were collected in the months of February, May–July and October–December, which, combined with the low total samples, excluded this species from examining the timing of opaque zone formation as was previously done for the north Caribbean (Shervette & Rivera Hernández, 2022a). For *B. capriscus*, the proportion of otoliths with opaque edges peaked from April to July (Figure 2). This information was combined with peak spawning period of *B. capriscus* in SEUS waters to establish a birthdate of 1 June so that fractional ages could be computed and utilized in the growth models for this species. Based on these results, the following rules were applied for computing fractional ages: (1) for fish caught February–May, with opaque zones on the edge, fractional age = opaque zone count – ((6 − month)/12); (2) for fish caught February–May with translucent zones on the edge, fractional age = (opaque zone count + 1) – ((6 − month)/12); (3) for fish caught June–December with either otolith edge type, fractional age = opaque zone count + ((month − 6)/12).

A total of 665 *B. capriscus* otoliths had two independent age estimates which resulted in an APE of 4.7%, perfect agreement for age estimates occurred for 62% of the samples, 85% had otolith age estimates within 1 year and 95% within 2 years. Analysis of between‐reader agreement for *B. veulta* otolith age estimates (APE = 3.6%) was previously described in Shervette and Rivera Hernández (2022a). Ten of the SEUS *B. veulta* otoliths were included as part of the APE calculation for the 510 otoliths with independent age estimates from the two readers.

**FIGURE 3:**
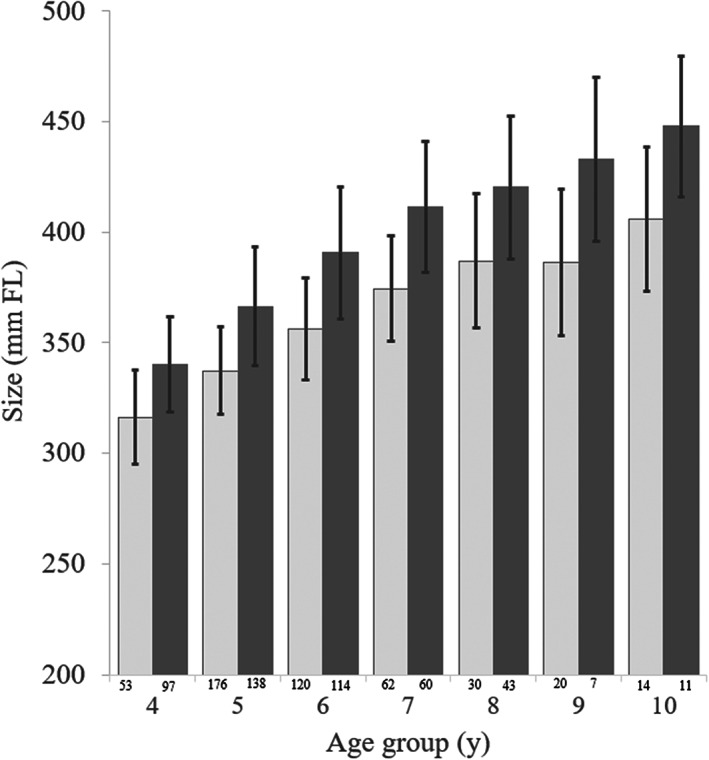
Mean size‐at‐age (± s.d.) comparison between male and female *Balistes capriscus* for ages 4–10 years). (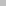) Female and (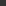) male. FL, fork length

*B. capriscus* males ranged in size from 26 to 571 mm *L*_f_ and in age from 0 to 17 years. Females ranged from 25 to 483 mm *L*_f_ and from 0 to 20 years (Table 1). Mean size at age of *B. capriscus* males and females differed significantly, with males larger than females in each of the age groups analysed (Figure 3 and Table 2). The size of *B. vetula* males ranged from 380 to 560 mm *L*_f_ and age ranged from 8 to 30 years. Female sizes ranged from 355 to 480 mm *L*_f_ and age from 8 to 40 years. Due to the small overall sample size of *B. vetula* collected within each age group from SEUS waters, we were unable to test for significant differences in size at age between the sexes as was previously documented for north Caribbean samples (Shervette & Rivera Hernández, 2022a).

**TABLE 2:** Summary of ANOVA results for differences in mean size‐at‐age between *Balistes capriscus* males and females

Source	Degrees of freedom	Sum of squares	Mean square	*F*	*P*
Size (*L*_f_ mm)					
Age (4–10 years)	6	589,539	589,539	150.6	<0.001
Sex	1	120,387	120,387	184.5	<0.001
Age × sex	6	2613	935	1.4	0.198
Error	931	607,390	652		

*Note*: *L*_f_, fork length.

**FIGURE 4:**
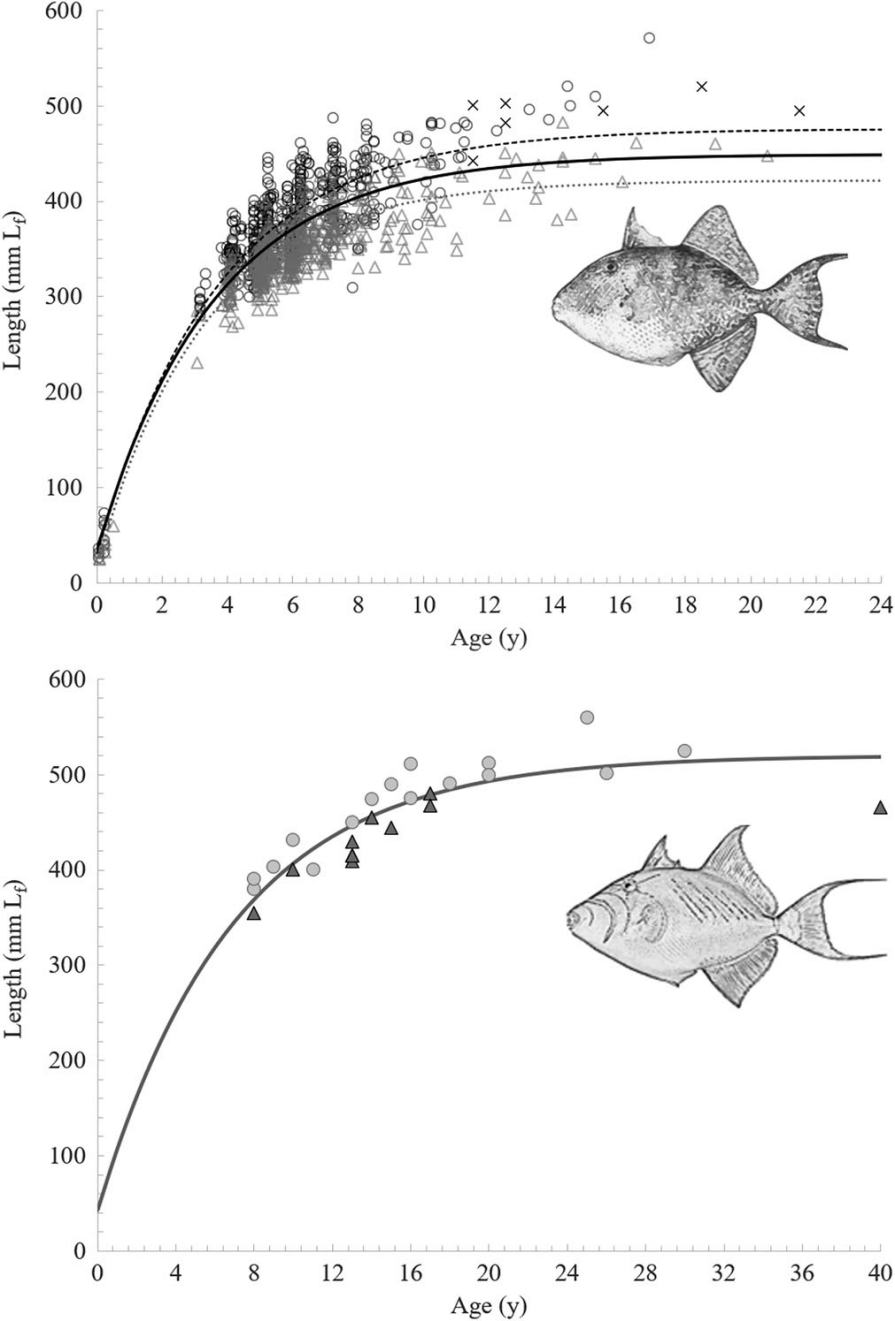
Length‐at‐age and von Bertalanffy growth function curves for *Balistes capriscus* (above) and *B. vetula* (below) samples from Atlantic waters of the south‐eastern United States. (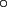) Male, (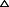) female, (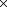) unknown, (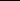) combined L_f_ = 449 [1 – e^−0.28(t+0.30)^], (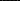) male L_f_ = 476 [1 – e^−0.27(t+0.26)^], and (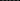) female L_f_ = 442 [1 – e^−0.29(t+0.19)^]. (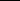) Combined L_f_ = 520 [1 – e^(−0.14(t+0.585))^], (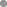) male and (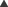) female. L_f_, fork length

The von Bertalanffy growth equation for all *B. capriscus* samples combined was *L*_f_ = 449 (1 − e^−0.28(*t*+0.30)^) (Figure 4 and Table 3). Using only female samples resulted in the following: *L*_f_ = 422 (1 − e^−0.29(*t*+0.19)^). For male *B. capriscus* the growth equation was *L*_f_ = 476 (1 − e^−0.27(*t*+0.26)^). *B. vetula* observed sizes at age yielded the following growth equation: *L*_f_ = 520 (1 − e^−0.14(*t*+0.585)^) (Figure 4 and Table 3). Note that a fixed *t*_0_ value was used for *B. vetula* due to a lack of fish <355 mm *L*_f_ (Shervette & Rivera Hernández, 2022b).

**TABLE 3:** Comparative summary of life‐history studies reporting on the von Bertalanffy growth function results for *Balistes capriscus* and *B. vetula* from spine‐based age estimation and otolith‐based age estimation that included small juvenile fish caught in pelagic habitat or newly recruited to benthic habitats

Species/region time period study	Structure (*n*); size range (mm *L*_f_); age range (years)	*L*_∞_ mm *L*_f_	*K*	*t*_0_	Source; gear; sampling design
*Balistes capriscus*					
NC‐SC 2012–2021 Current study	Otoliths (1044); 25–571; 0–21	All: 449 (441–458) M: 476 (463–489) F: 422 (414–431)	All: 0.28 (0.26–0.29) M: 0.27 (0.25–0.29) F: 0.29 (0.27–0.31)	All: −0.30 (−0.50 to −0.13) M: −0.26 (−0.46 to −0.09) F: −0.19 (−0.34 to −0.04)	FD + FI; opportunistic hook/dipnet; traps: random stratified
NC‐FL 2009–2012 Kelly‐Stormer *et al*. (2017)	Dorsal spine (1372); 26–523; 0–10	All: 382 M: 419 F: 352	All: 0.67 M: 0.54 F: 0.95	All: −0.47 M: −0.61 F: −0.22	FI; traps: stratified random
SC 2012–2014 Shervette *et al*. (2021)	Dorsal spine (642); 26–520; 0–10	All: 400	All: 0.63	All: −0.24	FD; opportunistic hook
North GOM 2003–2013 Allman *et al*. (2018)	Dorsal spine (5361); 3–697; 0–14	All: 484 M: 405 F: 387	All: 0.34 M: 0.55 F: 0.52	All: −0.06 M: 0.02 F: 0.004	FD + FI; hook, trap, spear, trawl; combination of stratified random and opportunistic
*Balistes vetula*					
NC‐SC 2013–2021 Current study	Otoliths (27); 355–560; 8–40	All: 520 (497–546)	All: 0.14 (0.12–0.17)	All: −0.585 (fixed)	FD; hook; opportunistic
Caribbean 2013–2021 Shervette and Rivera Hernández (2022b)	Otoliths (2045); 67–473; 0–23	All: 430 M: 441 F: 412	All: 0.15 M: 0.15 F: 0.15	All: −0.585 (fixed) M: −0.585 F: −0.585 (fixed)	FD + FI; hook/trap/spear; FI combination of opportunistic and stratified random FD random
Caribbean 2013–2021 Shervette and Rivera Hernández (2022a)	Dorsal spine (1622); 67–473; 0–14	All: 368	All: 0.34	All: −0.50 (fixed)	FD + FI; hook/trap/spear; FI combination of opportunistic and stratified random FD random

*Note*: Parameter estimates computed in the current study (*L*_∞_, *K*, *t*_0_) include 95% confidence intervals in parentheses. F, female; FD, fisheries‐dependent; FI, fisheries‐independent; *K*, growth coefficent; *L*_∞_, asymptotic size; *L*_f_, fork length; M, male; NC‐FL, North Carolina to Florida; NC‐SC, North Carolina to South Carolina; t_0_, theoretical age when size is zero; s.d., standard deviation.

## 4 DISCUSSION

The results from the current study provide critical new insights into the life history of two ecologically and economically important triggerfish species. This study is the first to report on sex‐specific age and growth information for *B. capriscus* using the Δ^14^C‐validated otolith‐based age estimation method. Our results indicate that the previous characterization of *Balistes* species as exhibiting moderately rapid growth and as relatively short‐lived, based on age estimates from the first dorsal spine, are flawed. Otolith‐based age estimates indicate that both *Balistes* species are relatively long‐lived, with maximum ages exceeding 20 years, and grow at slower rates than previously reported in spine‐based age studies. Future stock assessments for *Balistes* species should carefully evaluate the validity of life‐history parameter estimates derived from spine‐based ages and consider incorporation of otolith‐based age estimates for age‐related life‐history parameters such as longevity, growth, gear‐specific age at recruitment, mortality, maturity, and life‐time reproductive output.

### 4.1 Age and growth life‐history parameter estimates: otoliths versus spines

Many studies utilizing the first dorsal spine have noted the difficulty in age estimation for triggerfishes (Allman *et al*., 2018; Burton *et al*., 2015; Ingram, 2001; Kelly‐Stormer *et al*., 2017; Shervette & Rivera Hernández, 2022a). Thin sections of the dorsal spine do not present clear, easily discernable increments as evidenced by consistently low between‐reader precision (APE results ranging from 10% to 12%) reported across studies (Burton *et al*., 2015; Ingram, 2001; Kelly‐Stormer *et al*., 2017; Shervette & Rivera Hernández, 2022a). Additionally, recent Δ^14^C age estimation validation work indicated that triggerfish dorsal spines do not provide accurate age estimates, but otoliths do (Patterson *et al*., 2019; Shervette & Rivera Hernández, 2022a). Between‐reader precision results from otolith‐based age estimation from the current study for *B. capriscus* (APE = 4.7%), combined with precision results on otoliths of *B. vetula* (APE = 3.6%) (Shervette & Rivera Hernández, 2022a), indicate that otoliths appear to provide more precise age estimates. Triggerfish sagittal otoliths are small, fragile and can be difficult to extract intact consistently without proper training, but despite these challenges, otoliths do provide accurate and precise age estimates (Rivera Hernández & Shervette, 2022).

Direct comparisons of von Bertalanffy growth parameters among studies can be difficult and even inappropriate (Živkov *et al*., 1999) because of differences in the methods used to compute parameter estimates and differences in study design and sample collection methods. Therefore, we have limited our comparisons to past spine‐based age estimation studies that included juvenile triggerfish collected from pelagic habitat or that included newly recruited small fish to benthic habitat and produced biologically comparable *t*_0_ values and computed VBGF parameters from observed size‐at‐age data (Table 3). For *B. capriscus* the maximum spine‐based age from these studies was 14 years compared to a maximum otolith‐based age of 21 years (Table 3). The otolith‐based result extends *B. capriscus* longevity by approximately 30% in that this species can live up to 1.3 times longer than previously realized. The difference in longevity derived from spine‐based and otolith‐based ages is even more for *B. vetula*: otolith‐based age estimation extended maximum age from 14 to 40 years, indicating that this species can live up to three times longer than previously realized.

In the current study, *B. capriscus* otolith‐based age estimates from SEUS FD and FI samples yielded an asymptotic length (*L*_∞_) of 449 mm *L*_f_ [95% confidence interval (CI) 441–458] for all samples combined (Table 3), which was greater than the *L*_∞_ estimates from spine‐based ages from other SEUS studies (*L*_∞_ = 382–400 mm *L*_f_; Table 3). Otolith‐based ages resulted in a lower growth coefficient (*K* = 0.28, 95% CI 0.26–0.29; Table 3) than reported for spine‐based SEUS studies (*K* = 0.63–0.67; Table 3). However, the current study obtained otolith‐based age estimates from mainly FD samples from the SEUS and therefore may present a biased snapshot of growth (Taylor *et al*., 2005; Wilson *et al*., 2015) since FD samples would mainly represent fish caught *via* hook and line gear with relatively large hook sizes. Additional samples would be useful for SEUS *B. capriscus* from FI collections utilizing additional gears (*e.g*., chevron traps and dipnets) so that otolith‐based ages can be obtained and used to calculate VBGF parameters for a broader combination of FD and FI samples caught with a wider variety of gears.

One spine‐based age and growth study from the GOM, which included FD and FI *B. capriscus* from across multiple areas of the northern Gulf, obtained over 400 commercial long‐line samples that averaged 488 mm *L*_f_ (Allman *et al*., 2018). The combined VBGF that included those larger fish yielded *L*_∞_ = 484 mm *L*_f_ (Table 3). However, sex was not determined for the FD long‐line samples from that study because fish were eviscerated at sea (Allman *et al*., 2018). The mean size of GOM fish sampled from recreational hook‐and‐line was 365 mm *L*_f_, which is similar to the mean size of FD *B. capriscus* in the current study (369 mm *L*_f_, s.d. 43; Table 1). Although no sex information was available for the FD long‐line fish, Allman *et al*. (2018) obtained macroscopic sex information from 53% of their samples, ‘nearly all of which were recreational and fishery‐independent samples’. The otolith‐based VBGF results for males in the current study yielded a larger asymptotic size (*L*_∞_ = 476 mm *L*_f_, 95% CI 463–489) compared to males from Allman *et al*. (2018) (*L*_∞_ = 405 mm *L*_f_). Females in the current study had *L*_∞_ = 422 mm *L*_f_ (95% CI 414–431), also larger than what occurred in the GOM (387 mm *L*_f_) using spine‐based age estimates (Allman *et al*., 2018). The otolith‐based *K* for females and males (0.29 and 0.27; Table 3) were lower from the current study than the GOM spine‐based female and male VBGF results (0.52 and 0.55; Table 3). Otolith‐based ages of long‐line caught *B. capriscus* would be useful to obtain in future sampling efforts, as those larger fish may yield individuals that exceed the maximum age of 21 years found for *B. capriscus* in the current study, thereby potentially extending our understanding of longevity for this species.

*B. vetula* estimates of *L*_∞_ and *K* exhibited a similar pattern of differences: otolith‐based *L*_∞_ (430–520 mm *L*_f_) was larger than spine‐based *L*_∞_ (368 mm *L*_f_; Table 3). *B. vetula* otolith‐based age estimates from SEUS and U.S. Caribbean waters had similar *K* values (0.14 and 0.15, respectively) that were half of the *K* (0.34) reported from spine‐based ages (Table 3). However, the sample size of SEUS *B. vetula* from the current study was small, so additional samples are needed to provide a more comprehensive understanding of the potential differences in growth parameters for this species between the two regions.

Three clear trends for both *Balistes* species occurred when comparing otolith‐based VBGF estimates and longevity with those reported from spine‐based studies: *K* was consistently lower, asymptotic length was larger and maximum age was greater. These new insights combined with recent Δ^14^C triggerfish age validation results, that otoliths provide accurate ages and the dorsal spine does not (Patterson *et al*., 2019; Shervette & Rivera Hernández, 2022a), indicate that our past understanding of basic life‐history parameters for the two *Balistes* species was flawed. *B. capriscus* and *B. vetula* were previously described as moderately rapidly growing and relatively short‐lived (Burton *et al*., 2015; Manooch & Drennon, 1987) compared to other large‐bodied reef fishes that support fisheries in the western Atlantic, but based on our new understanding from otolith‐based ages we now know that *Balistes* species are moderately slow to slow growing and relatively long‐lived. Additional otolith‐based age sampling of *B. capriscus* in the SEUS and GOM is needed before additional stock assessments are conducted to ensure the accuracy of age‐based parameter estimates utilized in the stock assessment models. Spine‐based ageing methods do not appear to produce accurate ages, underestimate age (e.g., Figure 1c,d) and the resulting VBGF estimates do not reflect the same values as indicated by the results from our otolith‐based ages.

### 4.2 Age and growth life‐history parameter estimates: new insights

*B. capriscus* otolith edge analysis indicated that opaque zones, which represent periods of slower somatic growth, occurred mainly from spring to summer (April–July). This timing is in agreement with the *B. capriscus* spawning season (late April to early September; Kelly‐Stormer *et al*., 2017). During the spawning season, *B. capriscus* invest more energy into reproduction and less energy into somatic growth. A similar overlap in timing of *B. vetula* otolith opaque zone formation and peak spawning was observed in the Caribbean: otolith opaque zone formation occurred from December to March (Shervette & Rivera Hernández, 2022a) and peak spawning occurred from December to February (Rivera Hernández *et al*., 2019; Shervette & Rivera Hernández, 2022b). Other reef fishes in SEUS and Caribbean waters exhibit peak otolith opaque zone formation that overlaps with their reproductive season, including white grunt *Haemulon plumieri* (Lacepède, 1801) (Potts & Manooch, 2001), yellowtail snapper *Ocyurus chrysurus* (Bloch, 1791) (Garcia *et al*., 2003; Zajovits, 2021), snowy grouper *Epinephelus niveatus* (Valenciennes in Cuvier and Valenciennes, 1828) (Wyanski *et al*., 2000) and speckled hind *Epinephelus drummondhayi* (Goode and Bean, 1878) (Ziskin *et al*., 2011).

The maximum size of *B. capriscus* collected in the current study was similar to the maximum size from other SEUS studies. Samples from a study focused on FI fish collected from 1991 to 2012 *via* a combination of trap and hook gears had a maximum size of 578 mm *L*_f_ (Kelly‐Stormer *et al*., 2017). Another study from SEUS sampled *B. capriscus* caught with conventional hook gear from 1990 to 2012 commercial and recreational landings; the largest fish sampled was 567 mm *L*_f_ (Burton *et al*., 2015). The maximum size of *B. capriscus* included in a study on population demographics from SEUS waters was 585 mm *L*_f_ (Escorriola, 1991). *B. capriscus* population demographics studies from GOM waters have included larger maximum size fish than those from SEUS. A study targeting *B. capriscus* from the Alabama/Florida artificial reef zone had a maximum size of 617 mm *L*_f_ (Jefferson *et al*., 2019). Allman *et al*. (2018) had a maximum size fish of 697 mm *L*_f_ (Table 3), and Hood and Johnson (1997) included a maximum size fish of 725 mm *L*_f_; both of these large fish were caught using conventional hook gear. Federal and regional programs that reported on the size composition of *B. capriscus* from landings for the SEUS and GOM from 2011 to 2021 recorded maximum sizes of 644 and 633 mm *L*_f_ [NOAA Trip Interview Program (TIP, NOAA Southeast Fisheries Science Center), unpublished data], respectively, indicating that similar maximum sizes occur in the two regions. The lack of *B. capriscus* >600 mm *L*_f_ included in SEUS studies, combined with similar studies in GOM that consistently included fish >600 mm *L*_f_ and the fact that port sampling efforts have documented larger fish from SEUS waters, may indicate that these >600 mm *L*_f_ fish do occur in SEUS but may be rare. It is also possible that regional differences in the industry‐related fishing patterns of where fishers target *B. capriscus* along the continental shelf and slope depth gradient of the regional seascapes contributed to an undersampling of fish in the largest size groups for the SEUS. Allman *et al*. (2018) obtained the majority of *B. capriscus* in their study that exceeded 600 mm *L*_f_ from commercial long‐line landings (Table 3) which target fish in deeper shelf and slope waters of the GOM. *B. capriscus* population demographics studies from SEUS waters did not seem to include fish landed *via* long lines (Burton *et al*., 2015). Further investigation is needed to determine if differences exist in the maximum sizes of grey triggerfish between the two regions.

The maximum size of *B. vetula* sampled from SEUS waters in the current study was 560 mm *L*_f_, which is close to the maximum size of 585 mm *L*_f_ measured during 2012–2021 port sampling efforts in the North Carolina to South Carolina region (NOAA TIP, unpublished data). Recent life‐history work on *B. vetula* from the north Caribbean analysed FI and FD samples caught *via* a combination of gears including traps, spears and nets. The largest fish in the north Caribbean study was 473 mm *L*_f_. The largest fish sampled for another study in the area conducted from 1983 to 1984 was 419 mm *L*_f_ (Manooch & Drennon, 1987). Fish from the earlier study were caught mainly by trap gear. Gear selectivity may have played a role in the lack of larger fish for the two life‐history studies since larger *B. vetula* have reportedly been caught with hook gear in the north Caribbean (Stevens *et al*., 2019) and in the current study were landed in SEUS waters using hook gear.

The age and growth results from triggerfish otoliths indicate that the *Balistes* species had similar growth curve parameter estimates as those reported for several of the SEUS fisheries species in the same management complex that have been previously described as slow growing and long‐lived. As previously noted, care should be taken with making direct comparisons of growth parameter estimates among studies due to differences in study design and the methods used to calculate parameters. The growth coefficient for *B. capriscus* (*K* = 0.28) fell within the upper range of *K* reported for several snappers and groupers, while the growth coefficient of *B. vetula* (*K* = 0.14) was towards the lower end. Vermillion snapper *Rhomboplites aurorubens* (Cuvier, 1829) FD and FI samples combined from SEUS waters had a growth coefficient of 0.29 when *t*_0_ was constrained to −1.00 (SEDAR, 2008). Red grouper *Epinephelus morio* (Valenciennes in Cuvier and Valenciennes, 1828) and black grouper *Mycteroperca bonaci* (Poey, 1860) had estimated growth coefficients of 0.21 (*t*_0_ = −0.66) (SEDAR, 2010) and 0.17 (*t*_0_ = −0.77) (Crabtree & Bullock, 1998), respectively. Red snapper *Lutjanus campechanus* (Poey, 1860) from the south Atlantic region of the United States had a growth coefficient of 0.13 (*t*_0_ = −0.87) (White & Palmer, 2004) and gag grouper *Mycteroperca microlepis* (Goode and Bean, 1879) had a *K* of 0.24 (*t*_0_ = −0.48) (SEDAR, 2006).

*B. capriscus* and *B. vetula* maximum ages documented in the current study indicate that triggerfish species are long‐lived, with similar longevities to several of the SEUS grouper and snapper fisheries species. *B. vetula* attained a similar maximum age to what was reported for *L. campechanus* (*t*_max_ = 45 years) in the SEUS (White & Palmer, 2004), which is older than maximum reported ages documented for *E. morio* (*t*_max_ = 26 years), *M. microlepis* (*t*_max_ = 31 years) and *M. bonaci* (*t*_max_ = 33 years) from SEUS waters (Crabtree & Bullock, 1998; SEDAR, 2006, 2010). *B. capriscus* had a similar maximum age to *R. aurorubens* (*t*_max_ = 19 years) from the SEUS (SEDAR, 2008).

Some triggerfish species may be capable of plasticity in growth that is responsive to anthropogenic and environmental factors (Shervette *et al*., 2021). In the Gulf of Guinea, during a period of climatic shifts in oceanic attributes, the *B. capriscus* population experienced a rapid increase in abundance over a relatively short time span of 1972–1983 (Caverivière et al., 1980; Gerlotto, 2017). The rapid increase in abundance was correlated with an expansion of favourable environmental conditions for triggerfish from its normal benthic habitat to an additional suitable pelagic habitat (Gerlotto, 2017). For a fish population to expand from an estimated regional biomass of <1 *t* in 1972 to over 1,000,000 *t* by 1978, an increase in fish growth rate would seem to be a potentially important contributing factor (Shervette *et al*., 2021) combined with increased reproductive success. If growth in *B. capriscus* is relatively plastic, then it may vary temporarily and spatially in other regions of its range. Efforts to obtain otolith‐based age data for *B. capriscus* from contingents and populations across its range will be necessary to better understand the potential growth plasticity in this species.

Sex‐specific otolith‐based age estimation results from the current study, combined with similar results from the north Caribbean, confirm that *B. capriscus* and *B. vetula* are sexually dimorphic, with males attaining larger sizes‐at‐age compared to females. Previous investigations on *Balistes* species have noted that sexual dimorphism in triggerfishes relates to their reproductive strategy (Kelly‐Stormer *et al*., 2017; Rivera Hernández *et al*., 2019; Simmons & Szedlmayer, 2012). Triggerfishes have an ‘exceptional’ spawning strategy (Gladstone, 1994) in that they are relatively large‐bodied reef‐associated species that produce large amounts of eggs (Gladstone, 1994; Ingram, 2001; Kuwamura, 1997), but are benthic nesters that invest a large amount of energy in spawning territory defence and in brood care of their fertilized eggs (Fricke, 1980; Gladstone, 1994; Lobel & Johannes, 1980) to ensure that eggs successfully hatch into larvae that then move on to planktonic habitat (Kuwamura, 1997). Several studies on mating behaviour in triggerfishes have observed that the males defending nesting territories are larger than the females nesting within a territory (Fricke, 1980; Gladstone, 1994; Kuwamura, 1997; Seki *et al*., 2009; Simmons & Szedlmayer, 2012). A larger size for males may enhance their ability to successfully defend higher quality nesting territories from conspecifics and also enhance their success in attracting and mating with more females (Gladstone, 1994; Seki *et al*., 2009).

Mature females across a range of sizes utilize nests within a male territory and defend developing eggs from potential predators (Fricke, 1980; Gladstone, 1994; Kuwamura, 1997; Simmons & Szedlmayer, 2012). Female triggerfish invest a substantial amount of energy during their spawning season in nest preparation and maintenance, mating, tending to the fertilized eggs by fanning and blowing on them, and defending eggs from predators (Clark *et al*., 2015; Fricke, 1980; Gladstone, 1994; Kuwamura, 1997; Simmons & Szedlmayer, 2012). Females of several species do not appear to forage or exhibit reduced foraging efforts while caring for fertilized eggs compared to the effort they spend foraging outside of the nesting period (Fricke, 1980; Gladstone, 1994; Kuwamura, 1997). Females of several triggerfish species spawn multiple batches of eggs within a reproductive period (Gladstone, 1994; Kelly‐Stormer *et al*., 2017; Kuwamura, 1997; Rivera Hernández *et al*., 2019; Seki *et al*., 2009). The investment of energy by females into these reproductive activities for multiple broads each spawning season combined with reduced intake of food during that time may partially explain why females tend to be smaller than males since they are investing large amounts of energy in reproduction efforts and less energy during the spawning season in somatic growth. Further evidence of this substantial energy investment in reproduction and less in somatic growth for females from the two *Balistes* species comes from the maximum age results documented in the current study. The oldest *B. capriscus* in our study of known sex (20 years) was a 448 mm *L*_f_ female and the oldest *B. vetula* (40 years) was a 466 mm *L*_f_ female. Both of these females were in the upper size range for females of their species but were much smaller than the largest males from our study.

## 5 CONCLUSIONS AND MANAGEMENT IMPLICATIONS

King and McFarlane (2003) stressed that consideration of the life‐history strategies of species is fundamental to sustainable fisheries management because life‐history traits are the underlying determinants for fish population responses to anthropogenic and environmental forcing. Characterizing the life‐history traits of fish populations requires the application of accurate age estimation for managed species. *Balistes* triggerfish species in the Atlantic were previously described as moderately rapid growing and relatively short‐lived based on what we now know is an inaccurate age estimation structure/method. By obtaining age estimates from otoliths utilizing a bomb radiocarbon validated age estimation protocol, we have a new understanding of the life‐history traits of two important triggerfish species. *B. capriscus* and *B. vetula* are moderately slow‐growing and long‐lived species that exhibit a unique reproductive strategy involving substantial parental investment ensuring fertilized eggs successfully develop into larvae which may enhance life‐time reproductive output (another key characteristic that should be examined further for both species). Management efforts for triggerfishes should seriously evaluate these new insights on age and growth, and incorporate the results of otolith‐based age estimation into future population monitoring efforts.

## Supporting information


**Supporting Information Figure S1** *Balistes* triggerfish otolith illumination. Sagittal otoliths are read whole using a stereo microscope with reflected light against a black background while submerged in water at a magnification of 20–40×. (A) View of the mesial surface of a left sagitta illustrating the most relevant features related to enumerating opaque zones. Cauda is outlined with a solid black line and ostium is outlined with a dotted black line. Opaque zones are enumerated along the dorsal ridge of the cauda as indicated by the red zone. (B) Light intensity is key and should be adjusted until otolith opaque zones appear to glow. A fibre optic cable attached to the end of a light source can be used to effectively concentrate light and allow for light intensity control when visualizing opaque zones (shown here with a queen triggerfish otolith). (C) Example of the presentation of illuminated opaque zones of a grey triggerfish sagitta appearing to glow from concentrated light. (D) Example of the presentation of illuminated opaque zones of a queen triggerfish sagitta. (E, F) Note that the direction of growth for the ventral margin of the sulcus acusticus is such that the earliest increments are tucked down in the ‘funnelized’ cauda formation and to fully visualize this in otoliths with 20+ opaque zones requires gently tilting dorsal margin of the otolith towards the reader. (E) The ventral margin of the sulcus acusticus is outlined in green and the tip of the rostrum is indicated by the orange oval. The red line notes the dorsal edge of the cauda and represents the general region where we enumerate opaque zones. This otolith has 21 opaque zones. (F) The location of the ventral margin when this otolith only contained 20 opaque zones is indicated in green and the tip of the rostrum is indicated by the orange oval. The direction of growth is indicated with the black arrow, the current ventral margin is indicated by the yellow line and the red line notes the general region where we enumerate opaque zones. Note that the growth past 20 increments now obscures the path where we usually would enumerate the earliest opaque zones (dotted red line) so to visualize those, we would gently tilt the dorsal margin of the otolith towards the reader. This otolith has 40 opaque zones.
