# Supplementary material for: Illuminating otoliths: New insights for life history of Balistes triggerfishes
Source: J Fish Biol. 2022 Oct 18;101(6):1557–68. doi: 10.1111/jfb.15233 (PMC10092340; doi:10.1111/jfb.15233)
Supplement: Supplementary file 1 — Supporting Information Figure S1 Balistes triggerfish otolith illumination. Sagittal otoliths are read whole using a stereo microscope with reflected light against a black background while submerged in water at a magnification of 20–40×. (A) View of the mesial surface of a left sagitta illustrating the most relevant features related to enumerating opaque zones. Cauda is outlined with a solid black line and ostium is outlined with a dotted black line. Opaque zones are enumerated along the dorsal ridge of the cauda as indicated by the red zone. (B) Light intensity is key and should be adjusted until otolith opaque zones appear to glow. A fibre optic cable attached to the end of a light source can be used to effectively concentrate light and allow for light intensity control when visualizing opaque zones (shown here with a queen triggerfish otolith). (C) Example of the presentation of illuminated opaque zones of a grey triggerfish sagitta appearing to glow from concentrated light. (D) Example of the presentation of illuminated opaque zones of a queen triggerfish sagitta. (E, F) Note that the direction of growth for the ventral margin of the sulcus acusticus is such that the earliest increments are tucked down in the ‘funnelized’ cauda formation and to fully visualize this in otoliths with 20+ opaque zones requires gently tilting dorsal margin of the otolith towards the reader. (E) The ventral margin of the sulcus acusticus is outlined in green and the tip of the rostrum is indicated by the orange oval. The red line notes the dorsal edge of the cauda and represents the general region where we enumerate opaque zones. This otolith has 21 opaque zones. (F) The location of the ventral margin when this otolith only contained 20 opaque zones is indicated in green and the tip of the rostrum is indicated by the orange oval. The direction of growth is indicated with the black arrow, the current ventral margin is indicated by the yellow line and the red line notes the general reg [file JFB-101-1557-s001.pdf]

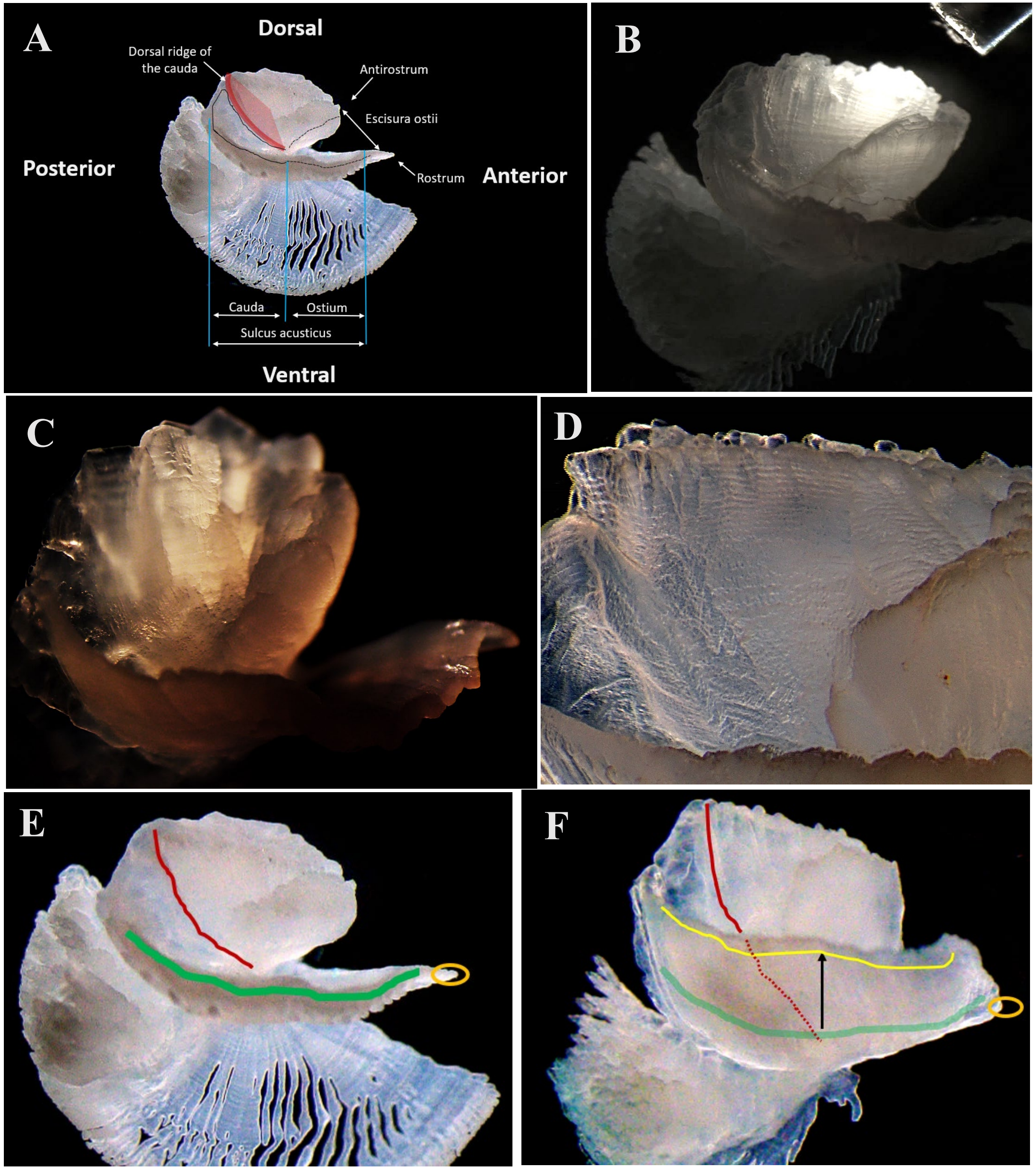

Figure 1 Supplement *Balistes* triggerfish otolith illumination. Sagittal otoliths are read whole using a stereo microscope with reflected light, against a black background while submerged in water at a magnification of 20-40x. (A) View of the mesial surface of a left sagitta illustrating the most relevant features related to enumerating opaque zones; cauda is outlined with a solid black line and ostium is outlined with a dotted black line; opaque zones are enumerated along the dorsal ridge of the cauda as indicated by the red zone. (B) Light intensity is key and should be adjusted until otolith opaque zones appear to glow; a fiber optic cable attached to the end of a light source can be used to effectively concentrate light and allow for light intensity control when visualizing opaque zones (shown here with a queen triggerfish otolith). (C) Example of the presentation of illuminated opaque zones of a grey triggerfish sagitta appearing to glow from concentrated light. (D) Example of the presentation of illuminated opaque zones of a queen triggerfish sagitta. (E-F) Note that the direction of growth for the ventral margin of the sulcus acusticus is such that the earliest increments are tucked down in the "funnelized" cauda formation and to fully visualize in otoliths with 20+ opaque zones, requires gently tilting dorsal margin of the otolith towards the reader. (E) The ventral margin of the sulcus acusticus is outlined in green and the tip of the rostrum is indicated by the orange oval; the red line notes the dorsal edge of the cauda and represents the general region where we enumerate opaque zones; this otolith has 21 opaque zones. (F) The location of the ventral margin when this otolith only contained 20 opaque zones is indicated in green and the tip of the rostrum is indicated by the orange oval; the direction of growth is indicated with the black arrow; the current ventral margin is indicated by the yellow line; the red line notes the general region where we enumerate opaque zones; note that the growth past 20 increments now obscures the path where we usually would enumerate the earliest opaque zones (dotted red line); so to visualize those, we would gently tilt the dorsal margin of the otolith towards the reader; this otolith has 40 opaque zones.
